# Cognitive Effects and Depression Associated With Taxane-Based Chemotherapy in Breast Cancer Survivors: A Meta-Analysis

**DOI:** 10.3389/fonc.2021.642382

**Published:** 2021-04-29

**Authors:** Eiman Y. Ibrahim, Ilaria Domenicano, Kate Nyhan, Mohamed Elfil, Sarah S. Mougalian, Brenda Cartmel, Barbara E. Ehrlich

**Affiliations:** ^1^Department of Pharmacology, Yale University, New Haven, CT, United States; ^2^Department of Biostatistics, Yale School of Public Health Yale University, New Haven, CT, United States; ^3^Harvey Cushing/John Hay Whitney Medical Library and Environmental Health Sciences, Yale School of Public Health, Yale University, New Haven, CT, United States; ^4^Department of Neurological Sciences, University of Nebraska Medical Center, Omaha, NE, United States; ^5^Department of Medicine (Medical Oncology), Yale School of Medicine, New Haven, CT, United States; ^6^Department of Chronic Disease Epidemiology Yale School of Public Health and the Yale Cancer Center, Yale School of Public Health, New Haven, CT, United States

**Keywords:** chemobrain, taxane, cognitive impairment, neuropsychology, neurophysiology, breast cancer

## Abstract

**Purpose:** This meta-analysis provides a longitudinal assessment of depression and cognitive impairment induced by taxane-based chemotherapy in women with breast cancer after 6 months of treatment. We highlighted the incidence and prevalence, the cognitive pattern in neuropsychological studies, and the relationship between chemotherapy-induced cognitive impairment and different risk factors. We estimated the effect sizes on each cognitive domain and differentiated effect sizes by each method of comparison of effects (i.e., baseline data, or control groups).

**Methods:** The databases MEDLINE and Embase were searched for publications about taxane-related cognitive changes in patients with breast cancer published from 1980 to 2019. Cross-sectional and self-reported outcomes studies were excluded except for the depression item. Included studies were assessed for risk of bias with the Newcastle–Ottawa Scale. We estimated effect sizes for each cognitive domain and differentiated effect sizes by each method of comparison of effects. The review is reported in compliance with the PRISMA Statement; it was registered prospectively in PROSPERO as CRD42020163255.

**Results:** Eleven studies meeting the criteria were analyzed, which resulted in a sample of 1,057 patients with breast cancer who received chemotherapy including 820 patients (77%) who received taxane-based chemotherapy. Attention and concentration, depression, and executive function domains had significant chemotherapy-induced impairment across all comparison types. Statistically significant improvement was found in language and verbal memory when comparing chemotherapy patients' test scores with baseline or matched controls. Taxane-based chemotherapy had a non-significant effect on processing speed, visual memory, visuospatial, and motor function domains.

**Conclusions:** The occurrence of chemotherapy-induced cognitive impairment 6 months or more after the course of treatment in people with breast cancer is frequent in the domains of attention, executive function, and depression. Other domains appear stable or improve with time after treatment cessation.

## Introduction

Breast cancer is the most common cancer affecting women in the USA. As of January 2020, there are more than 3.5 million breast cancer survivors in the USA, including patients currently being treated and survivors who have finished treatment (1). According to the National Cancer Institute definitions, “a person is considered to be a survivor from the time of diagnosis until the end of life” (2). Even in the era of immunotherapy and personalized medicine, chemotherapy is expected to continue to be a cornerstone in the adjuvant and neoadjuvant treatment settings, yet without treatment or prevention the debilitating side effects of chemotherapy remain (3). Up to 40% of all breast cancer survivors have lingering cognition-related complaints, sometimes known as “chemobrain” or “chemofog,” but more correctly designated chemotherapy-induced cognitive impairment (CICI). Patients typically demonstrate significantly lower scores in several cognitive tasks, including attention, memory, executive functions, and cognitive processing, when compared with subjects without cancer or cancer patients who did not undergo chemotherapy (4). Although underappreciated when chemotherapy was first used, healthcare providers are now acknowledging the high rate of patients who experience cognitive impairment and depression during and after chemotherapy and the impact of these changes on the patients' activities of daily life (5).

There is limited knowledge regarding CICI, and this is a consequence of several factors (6). Two major factors in this knowledge gap are inconsistencies and differences in the scales used to monitor cognitive side effect and the lack of sensitivity in the measurement scales. The majority of physicians and healthcare workers rely on patient self-reports to identify problems with cognitive functioning (6, 7). The measurement tools used for cognitive function have been developed to identify severe deterioration of mental fitness (6), as found in conditions such as Alzheimer's disease. This lack of sensitivity in the measurement means that loss of mental acuity in high functioning individuals will be missed (8). Together, these challenging factors lead to an uncertainty in evaluating the incidence of cognitive changes and diminish the urgency to address these side effects in this population of patients. It is clear that a therapeutic intervention is needed for this patient population to improve the general quality of life.

Previous meta-analyses that discussed CICI in women with breast cancer have claimed that the lack of consistency within the studies is a consequence of participant racial/ethnic diversity and socioeconomic status, treatment protocols, and variability in the neuropsychological tests used in assessments (9–12). Nevertheless, other than the impact of comparing patient performance with healthy controls or cancer patient controls, most of these meta-analyses have not systematically investigated the long-term effect of chemotherapy (≥6 months) on the various cognitive domains or examined the impact of one class of chemotherapy on the degree of observed cognitive impairments. Here, we provide a more focused analysis of the long-term effects of taxane-based chemotherapy on cognitive functions and depression in patients with breast cancer obtained by previous longitudinal studies. We used studies that monitored the nature and extent of cognitive impairments using objective neuropsychological assessments and excluded studies that depended on self-reports or one-time cross-sectional assessment except for the depression item as questionnaires and self-reports such as Beck Depression Inventory (13) and Hopkins Symptom Checklist (14) are well-validated and widely used as diagnostic modalities for depression.

CICI is a complex phenomenon influenced by underlying biological, neurological, and mental factors involving both unconscious and conscious psychological processes. Survivors describe cognitive changes such as memory lapses, learning difficulties, and troubles with focusing, planning, and multitasking that affect their personal and professional lives (15). Many factors contribute to the reported incidence of CICI including class of chemotherapy or other treatment received, duration of therapy, time since termination of chemotherapy, types of neuropsychological tests performed, and the definition of impairment used. Sometimes, these cognitive impairments are explained simply by the influence of the cancer diagnosis on mood status. Symptoms such as anxiety, with its subsequent impact on the capacity for attention, and depression are often associated with the unwanted diagnosis and associated chemotherapy (16).

Additional factors under-addressing the adverse cognitive events of chemotherapy include the lack of information underlying the etiology of CICI and the limited evidence for effective intervention strategies. Despite the high prevalence of cognitive impairment among cancer survivors treated with chemotherapy, there is significant variability in the way neuropsychological test scores are interpreted and reported (12, 17). Theories of “cognitive reserve” in highly educated and high functioning individuals may help explain the variance in neuropsychological tests interpretations (18, 19). For example, cognitive impairment was found to be more severe in groups with lower education, older age, non-white race, and metabolic comorbidities in a study that investigated cognitive reserve in 164 breast cancer patients and 182 controls (20). The test scores may also represent inherited rather than acquired abilities. Conversely, daily activities, level of education, and intelligence quotient seem to have a direct impact on cerebral blood flow and may modify cognitive reserve. These hypotheses suggest explanations not only for differences in neuropsychological performance but also for the susceptibility to chemotherapy-induced cognitive changes (19).

Efforts to identify and describe CICI have relied on several methods including neuropsychological assessments and self-reports, serum biomarker analysis, and imaging techniques such as structural and functional MRI (21). Comprehensive evaluations of cognitive functions can be achieved using a series of neuropsychological tests. However, these tests require special training for those performing and interpreting the tests and can be time-consuming (up to 6 h per test subject per session) making it unlikely that the tests will be universally used, particularly for patients dealing with cancer and its treatment (22). Instead, investigators have relied upon self-reported questionnaires in clinical trials to determine patients' cognitive impairment. However, many of these questionnaires have failed to show accuracy in both research and clinical settings (23). For these reasons, uniformly applied criteria, appropriate markers, and rapid quantitative tests are needed to build consensus on the incidence and impact of CICI.

This meta-analysis is focused on taxane (paclitaxel and docetaxel)–based chemotherapy because this class of chemotherapy is one of the most frequently used for the treatment of breast cancer (3) and other types of solid tumors (24, 25). Single agents including taxanes, anthracyclines, antimetabolites, and vinca alkaloids or combinations of these agents have demonstrated clinically significant favorable outcomes even in patients with metastatic breast cancer (26). Since the introduction of paclitaxel to the clinical use in mid-1990's, substantial efforts have been directed to study the adverse effects of this agent (27). Clinical studies and animal models have revealed significant deterioration of cognitive functions shortly after taxane-based chemotherapy (28–30). Few studies have assessed longitudinal changes of cognitive performance on cancer survivors after taxane therapy. The chemotherapeutic effect of taxanes is attributed to the stabilization of tubulin polymers causing mitotic arrest and apoptosis (31, 32). However, paclitaxel is responsible for several adverse effects particularly neuropathy, which appears to be independent of its effect on tubulin (33). Similar molecular changes on the central nervous system (CNS) have been suggested because taxanes can cross the blood–brain barrier and accumulate in the CNS (34). Furthermore, recent studies are designing treatments that improve delivery of paclitaxel into the brain to treat brain cancer (35, 36) supporting the need to better understand the toxic effects of taxanes on the CNS. We previously outlined a mechanism for paclitaxel-induced peripheral neuropathy (37) and our recent results using animal models to provide compelling evidence that similar mechanisms also underlie neuropathy in the CNS.

Although CICI is an existing, under-addressed phenomenon in breast cancer patients, vital questions concerning the course of cognitive changes related to taxane chemotherapy, the specific cognitive domains affected, and the underlying mechanisms remain unanswered. Without consensus on these questions, the best direction for treatment development is unclear. In this situation, a comprehensive synthesis of all published literature with summary statistics can provide useful information for defining the direction of future studies. Here, we aimed to conduct a systematic review with meta-analysis to focus on the association between taxane-based chemotherapy and cognitive impairment in women with breast cancer after 6 months of treatment.

## Methods

The protocol for this systematic review and meta-analysis was registered in the International Prospective Register of Systematic Reviews (PROSPERO) platform with the registration number of protocol ID: CRD42020163255 (38), and all the searches were posted on an Open Science Framework project (39). The protocol was drafted in accordance with the Preferred Reporting Items for Systematic Reviews and Meta-Analyses Protocols (PRISMA-P) statement guidelines and the Systematic Reviews and Meta-Analyses are reported based on the PRISMA guidelines (40, 41). The primary objectives of this review were to assess the effect of taxane-based chemotherapy on the cognitive function of patients with breast cancer and to summarize cognitive outcomes in breast cancer patients receiving taxane-based chemotherapy in terms of different functional domains.

### Searching Strategy

A comprehensive search was designed by a medical librarian (K.N.) and was peer-reviewed by an independent medical librarian. The search had three concepts, each one operationalized with both keywords and controlled vocabulary: cognitive function, breast cancer, and taxanes. Studies were retrieved from the two largest databases: MEDLINE All (through OvidSP) and Embase (through OvidSP). The search was peer reviewed by an independent medical librarian using the PRESS Peer Review of Electronic Search Strategies Guideline (42). The complete search strategy for MEDLINE was attached to our protocol registration. The complete search strategy for each database is presented in Appendix 1. The databases were searched in February 2020. Criteria for study population selection are presented in Appendix 2.

We systematically searched for eligible studies published from 1980, the year when studies showing cognitive impairments as a result of chemotherapy began to appear in the literature and taxanes started to be widely used as chemotherapy (5). We also set a language limit including only papers published in English. In addition, articles whose indexing indicates that they address only children were excluded. All records retrieved from the electronic searches in the databases were compiled, and duplicates removed, in Endnote X9. The records of included papers were reviewed to identify additional relevant papers.

### Study Selection

**Figure 1:**
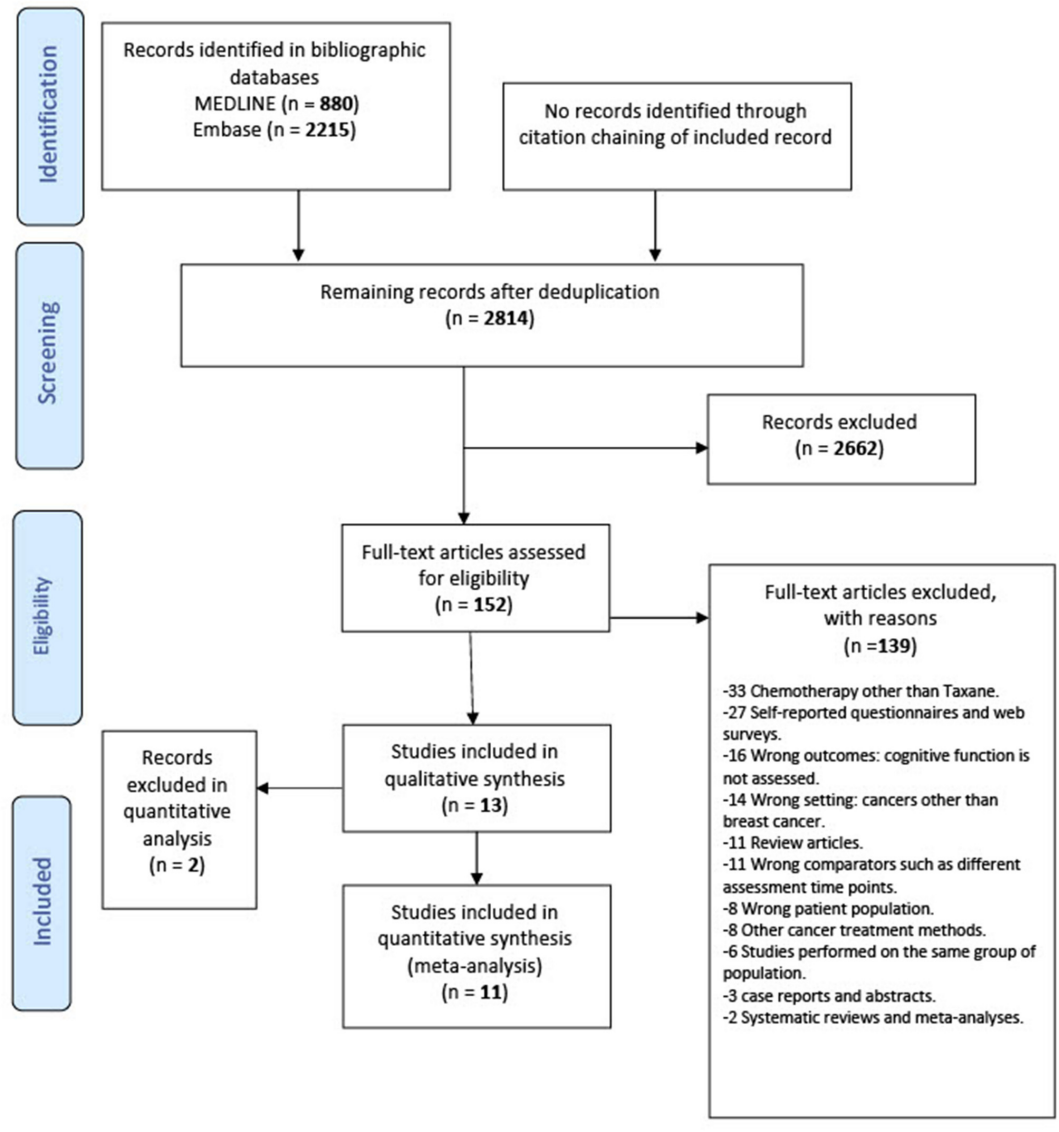
Preferred reporting items for systematic review and meta-analysis statement (PRISMA) flowchart of the included studies.

Two review authors (E.Y.I. and M.E.) independently screened titles and abstracts for relevancy in Covidence. In the full-text screening stage, the review authors inspected the full texts of potentially relevant records independently to judge on the eligibility using the inclusion and exclusion criteria. Studies were included in our systematic review if they fulfilled all the following criteria: cohort and case–control studies on adult patients with breast cancer, at any stage, with the exception of patients with brain metastases; minimum age 18 and maximum age 69; patients who had received taxane treatment (alone or in combination with other treatments); patients who were tested for any cognitive impairment following the taxane treatment of their breast cancer; patients who completed standard-dose taxane at least 6 months before assessment of cognitive impairment. Six months after treatment was chosen as a cutoff point to exclude assessment of the acute effects of chemotherapy (43). We excluded studies that included stage IV patients with brain metastasis due to the potential for direct effect of the tumor on the brain and consequently cognitive function. We also excluded research studies involving children, teenagers, and/or adolescents. For the meta-analysis of risk factors, we excluded studies that identified patients above 69 years of age because cognitive dysfunction can be more persistent among breast cancer with advanced age (44) and also to avoid any age-related neurodegeneration as a confounding factor. In case of conflicts, a third investigator (B.E.E. or B.C.) discussed and resolved the discrepancies. The flow of information from studies identified for inclusion followed the principles of the PRISMA Protocols (Figure 1).

### Data Extraction

For the eligible studies, two investigators (E.Y.I., M.E.) abstracted the data independently on a predefined agreed form using Covidence software. Data on authorship, publication year and study design, sample size age and education of the sample duration, and regimen of taxane therapy concurrent radiation or endocrine therapy, breast cancer stage, and neuropsychological test data were extracted from each study and are shown in Table 1. The individual neuropsychological measures assigned to each cognitive domain are shown in Table 2.

**Table 1:** Characteristics of included studies (*k* = 11).

**First author (year)**	**Country**	**Number received taxanes/total received chemotherapy**	**Number of controls**	**Cut off point assessment included in the analysis**	**Mean age of chemotherapy group (years)**	**Ethnicity and race of chemotherapy group**	**Mean years of education in chemotherapy group**	**Radiation therapy in chemotherapy group (*n*)**	**Endocrine therapy in chemotherapy group (*n*)**	**Number of postmenopausal subjects in chemotherapy group**	**Primary language of patients**	**Taxane chemotherapy regimen (*n*)**	**Study domains**	**Findings**
Ahles 2010 (45)	USA	24/60	72 no chemotherapy;45 healthy controls	Baseline and 6 months	51.7	White = 58, Asian = 1, Other = 0	15.7	49	39	32	English	Doxorubicin/cyclophosphamide/paclitaxel (22) Docetaxel/doxorubicin/cyclophosphamide (2)	Attention and concentration assessment, executive function, visual memory, verbal memory, processing of information, depression	Chemotherapy had a short-term effect on verbal ability. Age and cognitive reserve were related to decline in processing speed after exposure to chemotherapy
Cerulla 2019 (46)	Spain	25/51	N/A	Baseline and 1 year	51.5			47	47	23	Spanish	FEC and taxanes (25)	Attention and concentration assessment, executive function, visual memory, verbal memory, processing of information, motor function, visuospatial ability, depression	Progressive improvement over time in measures of memory and divided attention was observed when PE was not corrected. When PE was corrected, worsening was found in measures of memory, fluency, and executive function
Donovan 2005 (47)	USA	18/60	83	Baseline and 6 months	52.33	White = 52	14.97		31	33	English	Doxorubicin, cyclophosphamide, and docetaxel (6); doxorubicin, cyclophosphamide, and paclitaxel (10); and doxorubicin and docetaxel (2)	Attention and concentration assessment, executive function, visual memory, verbal memory, language	Women who underwent chemotherapy did not report significantly more problems with cognitive functioning than women treated without chemotherapy
Freeman 2002 (48)	USA	9	8 no chemotherapy	Baseline and 6 months	51.11		17.33				English	Doxorubicin and taxane	Attention and concentration assessment, language, verbal memory, motor function, visuospatial ability, depression	Chemotherapy group was significantly below the post-treatment group on the language and immediate memory subsets. They also showed a strong trend for slowed motor coordination
Janelsins 2018 (49)	USA	542/580	334 healthy controls	Baseline and 6 months	53.4	White = 517, African American = 47, Other = 16		285	171	303	No data	Paclitaxel *n* = 236; docetaxel *n* = 306	Attention and concentration assessment, executive function, verbal memory, processing of information, depression	CICI in patients with breast cancer affects multiple cognitive domains for at least 6 months post-chemotherapy
Jansen 2011 (50)	USA	49/71	N/A	Baseline and 6 months	50.8	White = 31, Asian = 29, African American = 5, Hispanic = 4, Other = 2	16.05		42	23 in taxane group, 13 in non-taxane group	English	49	Attention and concentration assessment, executive function, language, verbal memory, motor function, motor function visuospatial ability, depression	CICI appeared to be more acute than chronic side effects of therapy
Lyon 2016 (51)	USA	7175	N/A	Baseline, 6 months	51.52	White = 53, African American = 22		59	53	43	No data	Doxorubicin, cyclophosphamide and docetaxel *n* = 39; TC, docetaxel and cyclophosphamide *n* = 21; Docetaxel, carboplatin, and trastuzumab (herceptin) *n* = 11	Attention and concentration assessment, executive function, visual memory, verbal memory, motor function, depression	Most neuropsychologic scores improved over time, but memory did not improve after 2 years of chemotherapy cessation
Menning 2016 (52)	The Netherlands	24/31	24 no chemotherapy, 33 healthy controls	Baseline and 6 months	49.8			25	22	12	Dutch	AC+ docetaxel (21) AC + paclitaxel (3)	Attention and concentration assessment, executive function, language, processing of information, verbal memory, visual memory, motor function, depression	Women with breast cancer and systemic treatment had general worse performance compared with controls. Cognitively impaired patients had a significantly lower estimated baseline intelligence, worse physical and social functioning, and more distress compared with unimpaired patients
Shilling 2005 (53)	UK	6/50	N/A	Baseline and 6 months	51.1		11.96			24	English	FEC+ docetaxel (6)	Attention and concentration assessment, executive function, processing of information, verbal memory, visual memory	Women with breast cancer and systemic treatment were more likely to show cognitive decline than controls. They also showed significant increases in endocrine symptoms and fatigue post-treatment
Wefel 2010 (43)	USA	34/42	N/A	Baseline and 13 months after initiation of chemotherapy	48.8	White = 31; African American = 4, Hispanic = 7	13	22	13	21	English	FAC paclitaxel (34)	Attention and concentration assessment, executive function, processing of information, verbal memory, depression	Systemic chemotherapy is associated with decline in cognitive function during and shortly after completion of chemotherapy (61% of patients). Delayed cognitive dysfunction occurred in 29% of patients
Yao 2017 (54)	Canada	28/28	16	Baseline and 9 months	45.3				19		No data	AC–paclitaxel (4), FEC–docetaxel (20), AC–docetaxel(2), TCH (1), unknown = 1	Visual memory, language, and visuospatial ability	Women with breast cancer deteriorated in attention and inhibitory control relative to their pretreatment performance

*FAC, fluorouracil–adriamycin–cyclophosphamide; FEC, 5 fluorouracil–epirubicin–cyclophosphamide; TCH, taxotere (docetaxel) + carboplatin + herceptin; AC, adriamycin–cyclophosphamide*.

**Table 2:** Cognitive domains assigned to the neuropsychological measures.

**Attention and concentration assessment**
TMT-A Trail Making Test
Digit Span of the Wechsler Adult Intelligence Scale (WAIS)-III
Trail Making Test TMT A and B
Distractibility. Continuous Performance Test (CPT)
CNS Vital Signs: Stroop (correct responses)
Continuous Performance (correct responses)
RBANS
**Executive function**
COWA
The Trail Making Test (TMT-B)
The Stroop task
Shifting Attention (correct responses)
WAIS-III Letters and Numbers sequencing
Verbal ability. Vocabulary [WASI, Verbal Fluency Test (Delis–Kaplan Executive Function System (D-KEFS))]
**Depression**
Beck Depression Inventory
Hopkins Symptom Checklist
CES-D, Center for Epidemiological Studies-Depression
Hospital Anxiety and Depression Scale (HADS)
Center for Epidemiological Studies-Depression (CES-D) scale
Multidimensional Fatigue Symptom Inventory
Self-report measures of depression (Center for Epidemiological Study)
Depression, anxiety (Spielberger State Anxiety Inventory)
Multiple Ability Self-Report Questionnaire
**Language**
Controlled Oral Word Association from the Multilingual Aphasia Examination (COWA)
Boston Naming Test
Patient's Assessment of Own Functioning Inventory (PAOFI)
RBANS Language
**Processing of information**
WAIS-R Digit Symbol
TMT-A
Letter cancellation task
WAIS-III Digit Symbol
CNS Vital Signs: Symbol digit coding
Color-Word Interference Test (D-KEFS)
Grooved Pegboard
**Verbal memory**
HVLT total
HVLT immediate recall
WMS logical memory, immediate, and delayed
AVLT recall 1–5
Buschke Selective Reminding Test
RBANS
The California Verbal Learning Test (CVLT)
Wechsler Memory Scale (WMS–III)
California Verbal Learning Test-II
**Visual memory**
WMS immediate recall
Benton Visual Retention Test
Complex Figure, copy, immediate and delayed recall
Patient's Assessment of Own Functioning Inventory (PAOFI)
The Visual Reproduction of the Wechsler Memory Scales-III (WMS-III)
Faces I and II (WMS-III)
Rey–Osterrieth Complex Figure Test (ROCFT)
**Visuospatial**
Rey Complex Figure Test
RBANS Visual Construction
Benton Judgment of Line Orientation (JLO)
**Motor function**
Finger Tapping dominant hand
Grooved Pegboard for motor functioning
CNS Vital Signs: finger tapping

Due to limited data on assessment of the taxane effect on cognitive impairment in breast cancer patients, we included every possible study that contained taxane in the treatment course of breast cancer patients including both longitudinal cohort and case–control studies and we aimed at illustrating the mean sample of breast cancer patients who are being treated with taxane and highlight the cognitive impairment domains affected by chemotherapy. Subsequently, we performed a sub meta-analysis including only cohort studies and excluding the case–control studies to avoid any confounding results that could be induced by the manifestation of breast cancer itself when comparing patients with breast cancer with healthy controls.

We also extracted data from the studies of Tager et al. (55) and Thornton et al. (56), but we were not able to include these data in the quantitative synthesis for the meta-analysis purposes. Tager et al. (55) studied the cognitive effects of chemotherapy in post-menopausal women before adjuvant therapy, 6 months after treatment along with another 6-month follow-up assessment. They compared breast cancer patients who underwent chemotherapy with another group of breast cancer patients who did not receive chemotherapy. However, the published data only reported the baseline characteristics between the two groups and the data did not significantly differ on any test or domain. In addition, they did not report the changes over the two assessment times. Thornton et al. (56) investigated mental health outcomes in breast cancer patients who received taxane-based adjuvant chemotherapy in comparison with patients who did not receive taxanes. However, the study lacked any details on the neurophysiological assessments in terms of the different cognitive function domains.

### Risk of Bias and Quality Assessment

At least two investigators (selected from E.Y.I., M.E., and I.D.) independently assessed each eligible study for methodological quality using the Newcastle–Ottawa scale for cohort and case–control studies. The Newcastle–Ottawa quality assessment scale provides a maximum of nine points for the least risk of bias in three domains: (a) selection of study groups (four stars), (b) comparability of groups (two stars), and (c) assessment of outcome measures (three stars) for case–control and cohort studies (57). Any disagreement was resolved by a third reviewer (B.E.E.).

### Data Synthesis and Analysis

The main outcomes of interest for this analysis were eight cognitive domains: attention and concentration, executive function, language, visual memory, verbal memory, processing of information, and visuospatial and motor function. In addition, we suggested to study depression as it significantly contributes to our understanding of cognition particularly in patients with cancer (58). Visuospatial ability was only discussed in two studies (48, 50). We decided to combine motor function with visuospatial as a new domain which is different than motor functions only domain. After extracting the data from the included studies, we used Cohen's d effect size to estimate the effect sizes and the 95% CIs (59). Pooled variances were used for the estimation of the effect sizes to avoid potential bias arising from studies with small sample sizes. When a study reported multiple tests to measure the same cognitive domain, we computed an average effect size for that domain. Fixed-effects and random-effect meta-analyses were performed based on estimates of effect sizes and their standard errors. The inverse variance weighting method was used for pooling the effect sizes.

Effect sizes were interpreted as insignificant if they were <0.20, small if they were 0.20–0.50, medium if they were 0.50–0.80, and large if they were >0.80. A significance level of 0.05 was inferred when the 95% CI interval did not cross zero (59).

For neurological domains such as attention and concentration, executive function, language, visual memory, verbal memory, processing of information, and visuospatial and motor function, effect sizes were coded so that positive scores indicated better cognitive function and negative scores indicated worse cognitive function in the chemotherapy group. Conversely, a positive score for the domain related to the depression indicated an impairment in the chemotherapy group.

We assessed statistical heterogeneity using the I^2^ statistics and we tested for reporting biases such as publication bias using funnel plots. Statistical analyses were conducted through the statistical software R 4.0.1. The R package “meta” was used to perform the meta-analyses. However, the expected effect sizes heterogeneity cannot easily be achieved through existing fixed-effects methods. In response, we used a random-effect model for the meta-analysis with flexible expected variations across the studies (60, 61).

## Results

### Study Selection

Thirteen studies were identified for inclusion in this review. The searches of MEDLINE and Embase retrieved a total of 3,095 records. After deduplication, 2,814 records remained. Of these, 2,662 records were discarded as a result of title-abstract screening because they clearly did not meet the inclusion criteria. The remaining 152 records were assessed for eligibility in full-text screening. Thirteen studies met the inclusion criteria and were included in the systematic review. The cited references in these included studies were reviewed, but no additional studies were identified. Of the 13 included studies, 11 were suitable to include in the quantitative meta-analysis (Figure 1).

### Quality Assessment

**Figure 2:**
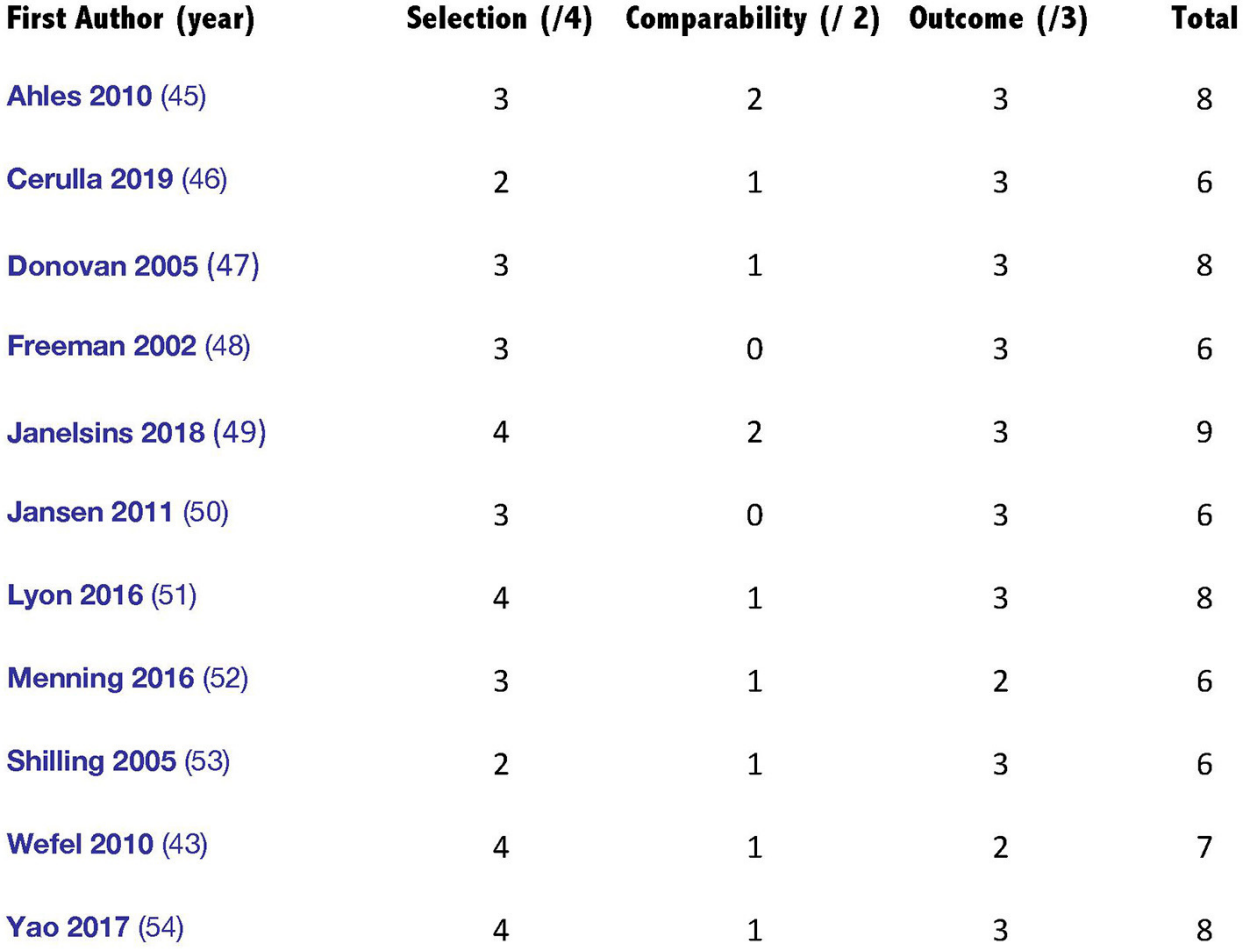
Summary of critical appraisal of included studies using the Newcastle–Ottawa Scale (NOS).

The Newcastle–Ottawa Scale (NOS) shows that the 11 included studies had a median score of seven, with a maximum score of nine and a minimum of six (57). Three cross-sectional studies and the three case–control studies included in the current review were of relatively low to moderate quality. In total, all the included studies had a good or excellent methodological quality. Figure 2 illustrates the assessment scores of the included studies.

### Baseline Study Characteristics

The studies comprised a sample of 1,057 breast cancer female patients who received chemotherapy, the majority of whom (820 patients 77%) received taxane-based chemotherapy. The mean age of those who received chemotherapy was 50.7 years and 537 (51%) subjects were postmenopausal. Of studies that reported endocrine and radiation therapy, 437 (43%) patients received endocrine therapy and 487 (58%) received radiation treatment. Of the six studies reporting education, we found that the mean years of education was 14.8. In the studies that reported race and ethnicity, 83.6% of studies participants were White, 8.8% were Black, and 7.6% were from other racial groups. In addition to the main taxane-based chemotherapy group, we also included two comparison groups, namely, patients with breast cancer who did not undergo chemotherapy and healthy control group.

### Meta-Analysis

Due to the inconsistencies of the used assessment models in literature, the use of a single, universal unit (e.g., effect size) was relied on to generate findings and form a consensus on the effects of taxane-based chemotherapy on cognitive functioning among breast cancer patients. Unweighted average effect sizes for each cognitive domain are shown in Table 3. Cognitive function assessed 6 months or more post-completion of chemotherapy was compared with baseline cognitive function in 81% of included studies (*K* = 9), with a healthy control group in 36% of included studies (*K* = 4), and with breast cancer patients who had not received chemotherapy in 45% of included studies (*K* = 5) (Table 3).

**Table 3:** Unweighted effect sizes (Cohen's D) for individual studies by type comparison and cognitive domain.

	**Attention and concentration**	**Depression**	**Executive function**	**Processing speed**	**Verbal memory**	**Visual memory**	**Motor function**	**Language**
**Chemotherapy vs. non-chemotherapy**								
Menning	−0.48	0.24	−0.38	−0.17	−0.58	0.21	0.42	–
Yao	–	–	–	–	–	0.69	0.69	0.31
Freeman	−1.15	0.75	–	–	0.72	–	0.54	0.74
Donovan	−0.00	–	0.15	–	0.04	0.29	0.22	−0.18
Ahles	0.20	–	0.10	0.15	0.23	0.18	–	–
**Chemotherapy vs. baseline**								
Wefel	0.40	–	0.08	0.64	−0.51	–	–	–
Menning	−0.03	−0.15	−0.03	0.09	−0.32	−0.44	0.15	–
Shilling	0.16	–	0.15	0.33	−0.11	0.41	–	–
Yao	–	–	–	–	–	0.35	0.82	0.61
Lyon	0.93	0.44	1.39	–	0.85	−0.61	0.58	–
Jansen	0.00	–	0.77	–	−0.26	–	−1.24	0.95
Janelsins	−2.0	–	−1.07	–	6.6	–	–	–
Cerulla	−0.06	0.31	−0.04	0.01	0.53	0.40	0.30	–
Ahles	0.18	–	0.25	0.19	0.89	1.31	–	–
**Chemotherapy vs. healthy control**								
Shilling	−0.41	–	−0.33	−0.37	−0.32	−0.00	–	–
Janelsins	0.50	–	−0.11	–	1.03	–	–	–
Menning	−0.12	0.77	−0.42	−0.26	−0.02	−0.16	0.17	–
Ahles	−0.61	–	−0.14	−0.47	−0.18	−0.1	–	–

Included in the meta-analysis are published longitudinal studies that examined multiple cognitive domains to define a stratum of breast cancer patients at high risk of CICI. Here, we included studies that assessed cognitive function in patients who received taxane treatment at the 6-month time-point.

Most studies reported multiple cognitive domains and time-points for each cognitive function. Eight of the 11 studies assessed cognitive function 6 months after completion of chemotherapy; of the remaining three, assessments were made 9 months (54), 1 year after completion of chemotherapy (46), and 13 months after initiation of chemotherapy (43). We did not conduct a separate analysis for the studies that reported on assessment of cognitive function at more than one time-point. Instead, we conducted an additional analysis that included assessments of the 6-month time-point or the closest to it (Tables 4, 5). The eight cognitive domains were not assessed in every study; the number of domains included in each study is also shown in details in Tables 4, 5. Studies reporting the same outcomes for different comparator groups (healthy control or baseline and breast cancer patients not receiving chemotherapy) were analyzed separately because of the importance of the differences between the comparison groups.

**Table 4:** Collective results for cognitive domain by comparison method using fixed effect model (in bold *p* < 0.005).

**Comparison group/time-point**		**Attention and concentration**	**Depression**	**Executive function**	**Processing speed**	**Verbal memory**	**Visual memory**	**Motor function**	**Language**
Baseline	Number of studies	8	3	8	5	8	6	5	2
	SMD	**−0.89**	**0.28**	**−0.30**	**0.23**	**1.46**	**0.18**	0.15	**0.85**
	95% CI lower	**−0.98**	**0.06**	**−0.41**	**0.04**	**1.33**	**0.02**	−0.03	**0.55**
	95% CI upper	**−0.77**	**0.50**	**−0.20**	**0.42**	**1.60**	**0.35**	0.34	**1.14**
		0.98	0.47	0.97	0.10	0.99	0.91	0.92	0.02
Healthy control	Number of studies	4	1	4	3	4	3	1	0
	SMD	**0.28**	**0.77**	**−0.15**	**−0.38**	**0.74**	−0.05	0.17	–
	95% CI lower	**0.16**	**0.26**	**−0.27**	**−0.63**	**0.61**	−0.30	−0.31	–
	95% CI upper	**0.40**	**1.28**	**-0.03**	**-0.12**	**0.86**	0.20	0.66	–
		0.92	–	0.00	0.00	0.95	0.00	–	–
No chemotherapy	Number of studies	4	2	3	2	4	4	4	3
	SMD	−0.06	0.36	0.04	0.05	0.04	**0.29**	**0.35**	−0.00
	95% CI lower	−0.28	−0.11	−0.18	−0.25	−0.17	**0.07**	**0.108**	−0.28
	95% CI upper	0.15	−0.82	0.26	0.35	0.26	**0.50**	**0.60**	0.27
		0.65	0.00	0.34	0.00	0.62	0.00	0.00	0.53

*SMD, standardized mean difference*.

**Table 5:** Results from random-effect meta-analysis for cognitive domain by comparison method (in bold *p* < 0.005).

**Method**		**Attention and concentration**	**Depression**	**Executive function**	**Processing speed**	**Verbal memory**	**Visual memory**	**Motor function and visuospatial ability**	**Language**
Baseline	Number of studies	8	3	8	5	8	6	5	2
	SMD	−0.05	0.24	0.18	**0.24**	0.95	0.23	0.12	**0.84**
	95% CI lower	−1.0	−0.06	−0.51	**0.03**	−0.95	−0.35	−0.56	**0.54**
	95% CI upper	0.89	0.56	0.88	**0.43**	2.87	0.82	0.80	**1.14**
Healthy control	Number of studies	4	1	4	3	4	3	1	0
	SMD	−0.14	**0.77**	–**0.15**	–**0.38**	0.14	−0.05	0.17	–
	95% CI lower	−0.76	**0.26**	–**0.27**	–**0.63**	−0.68	−0.3	−0.31	–
	95% CI upper	0.47	**1.28**	–**0.03**	–**0.13**	0.96	0.2	0.66	–
No chemotherapy	Number of studies	4	2	3	2	4	4	4	3
	SMD	−0.19	0.36	0.02	0.05	0.03	**0.29**	**0.35**	0.15
	95% CI lower	−0.61	−0.11	−0.26	−0.25	−0.36	**0.07**	**0.10**	−0.36
	95% CI upper	0.22	0.83	0.30	0.35	0.43	**0.50**	**0.60**	0.66

### Results Using Fixed-Effect Model

**Figure 3:**
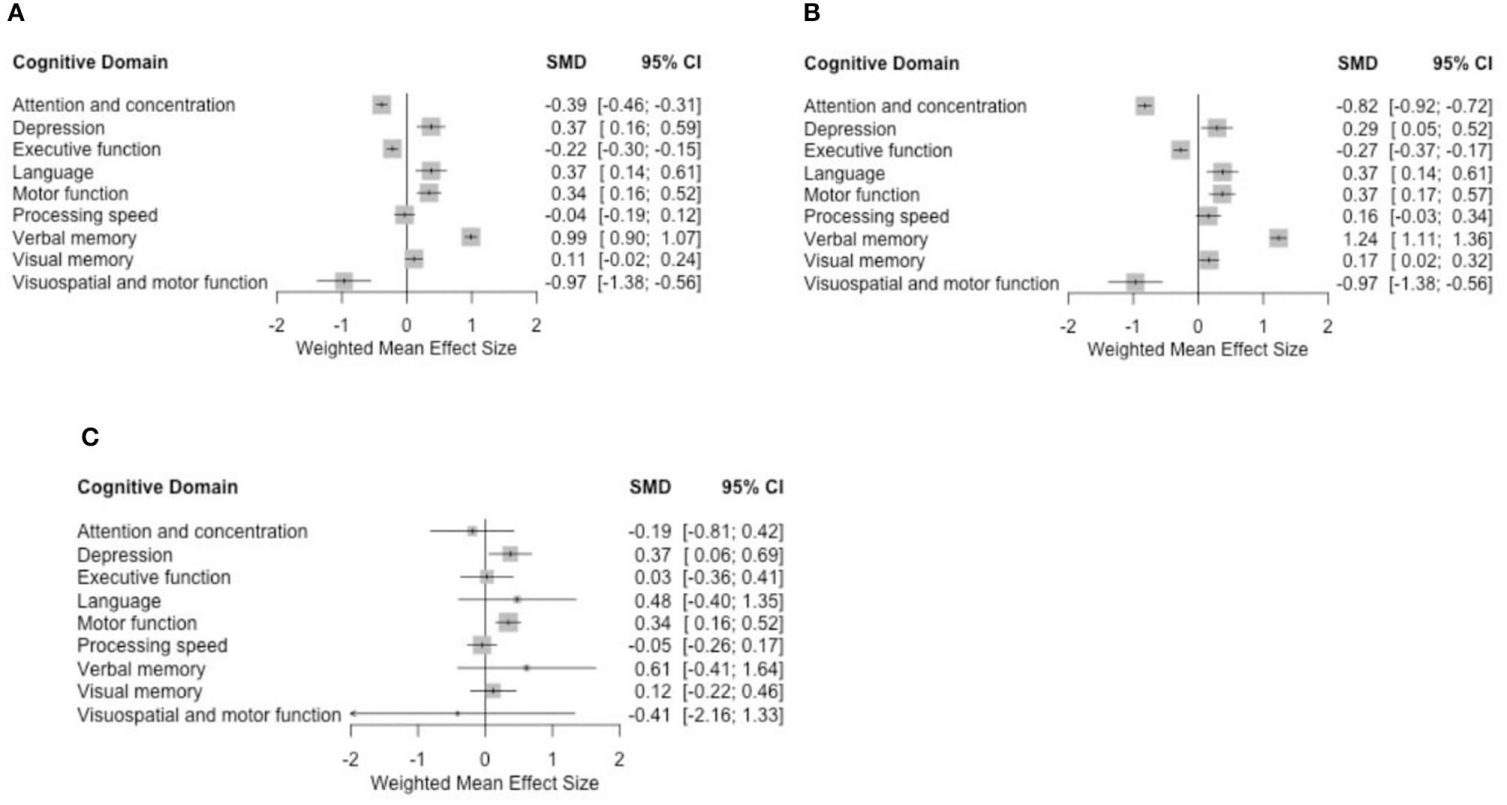
Forest plots using **(A)** fixed-effect model for all studies, **(B)** fixed-effect model for cohort studies only, and **(C)** random-effect model for 6-month time-point analyses.

1. Assessment at the 6-month post-chemotherapy completion (6MPCC) time-point
  Data pooled from the eight studies showed a significant association between taxane-based chemotherapy and the deterioration of attention and concentration, executive function, visuospatial ability, and depression (in the depression case, higher scores indicate more severe condition) with standardized mean difference (SMD) = −0.39 (−0.46, −0.31), −0.22 (−0.3, −0.15), −0.97 (−1.38, −0.56), and 0.37 (0.16, 0.59), respectively, p < 0.005 in all these findings. Visuospatial ability was only discussed in two studies (48, 50). When motor function was evaluated with visuospatial ability, we observed an impairment. Our results (Figure 3A) do not provide significant evidence for changes to processing speed, visual memory, and motor function domains. However, we found a positive correlation between taxane-based treatment and the improvement in language and verbal memory domains SMD = 0.37 (0.14, 0.61) and 0.99 (0.9, 1.07), *p* < 0.005 in both findings.
2. Collective analyses for 6-month and more time-points
  Analyses were then undertaken for all patients, including different comparison groups, either healthy controls, patients with breast cancer without chemotherapy, or their own baseline performance. As shown in Table 4, for studies that compared long-term neuropsychological test scores of breast cancer patients who received chemotherapy with their baseline data, patients treated with chemotherapy displayed significantly worse cognitive functioning in the domains of attention and concentration, executive function, and depression. Also, statistically significant moderate effect sizes were found in executive function, processing speed, and depression in studies that compared breast cancer chemotherapy group with healthy controls (*p* < 0.005). Patients treated with taxane-based chemotherapy tended to have better scores in processing speed, verbal, and visual memory as well as language domains when compared with their baseline assessment and in visual memory, visuospatial ability, and motor functions when compared with patients with breast cancer who have not received chemotherapy. Despite the negative trend in neuropsychological measures in the domains of attention and concentration, executive function, processing speed, and visual memory, our analysis failed to find a statistically significant impact of taxane-based chemotherapy on these domains when compared with breast cancer patients who have not received chemotherapy.
3. Assessment at the 6-month post-chemotherapy completion (6MPCC) time-point in cohort studies only
  Data pooled from the seven longitudinal cohort studies showed a statistically significant association between taxane-based chemotherapy and the deterioration of attention and concentration, executive function, visuospatial ability, and depression SMD = −0.82 (−0.92, −0.72), −0.27 (−0.37, −0.17), −0.96 (−1.37, −0.55), and 0.28 (0.05, 0.52), respectively. We also found statistically significant improvement in motor function, visual memory, and language SMD = 0.36 (0.17, 0.56), 0.16 (0.01, 0.31), and 0.37 (0.13, 0.60), respectively, with *p* < 0.005 in all previous findings (Figure 3B).

### Results Using Random-Effect Model

Due to study heterogeneity, we also assessed the outcomes using a random-effect model in all comparisons. For random-effect models, it is presumed that the true effect size differs across studies due to known and unknown reasons (such as subjects' characteristics, cognitive domains assessed).

1. Assessment at the 6-month post-chemotherapy completion (6MPCC)

Overall mean effect sizes after using the random-effect model showed a non-statistically significant negative effect on attention and concentration, processing speed, and visuospatial ability. Depression remained significantly affected. Improvement was observed in the language and verbal memory domains with additional improvement in the motor function. These results are consistent with the fixed-effect model analysis (Figure 3C).

2. Collective analyses for the 6-month and more time-points

No statistically significant negative effects were shown on cognitive function in the studies comparing taxane-based chemotherapy with the baseline time-point. We only found statistically significant improvements in language and processing of speed (Table 5). Visual memory, and visuospatial and motor function domains also were improved when compared with healthy controls. In addition, when applying a random-effect model on studies comparing taxane-based chemotherapy patients with healthy controls, we found a general negative effect of chemotherapy on cognitive function, primarily evident in depression, executive function, and processing of speed domains (Table 5).

The original plan was to include a sub-analysis of (1) the relationship between doses of taxanes and the deterioration of cognitive status, (2) number of patients who had to discontinue the treatment due to the cognitive side effects, and (3) the relationship between a certain comorbidity and additional comorbidities such as obesity and heart disease. These planned subgroup analyses could not be performed due to insufficient data and lack of research on these aspects. To our knowledge, there were no published reports discussing these issues.

### Publication Bias

**Figure 4:**
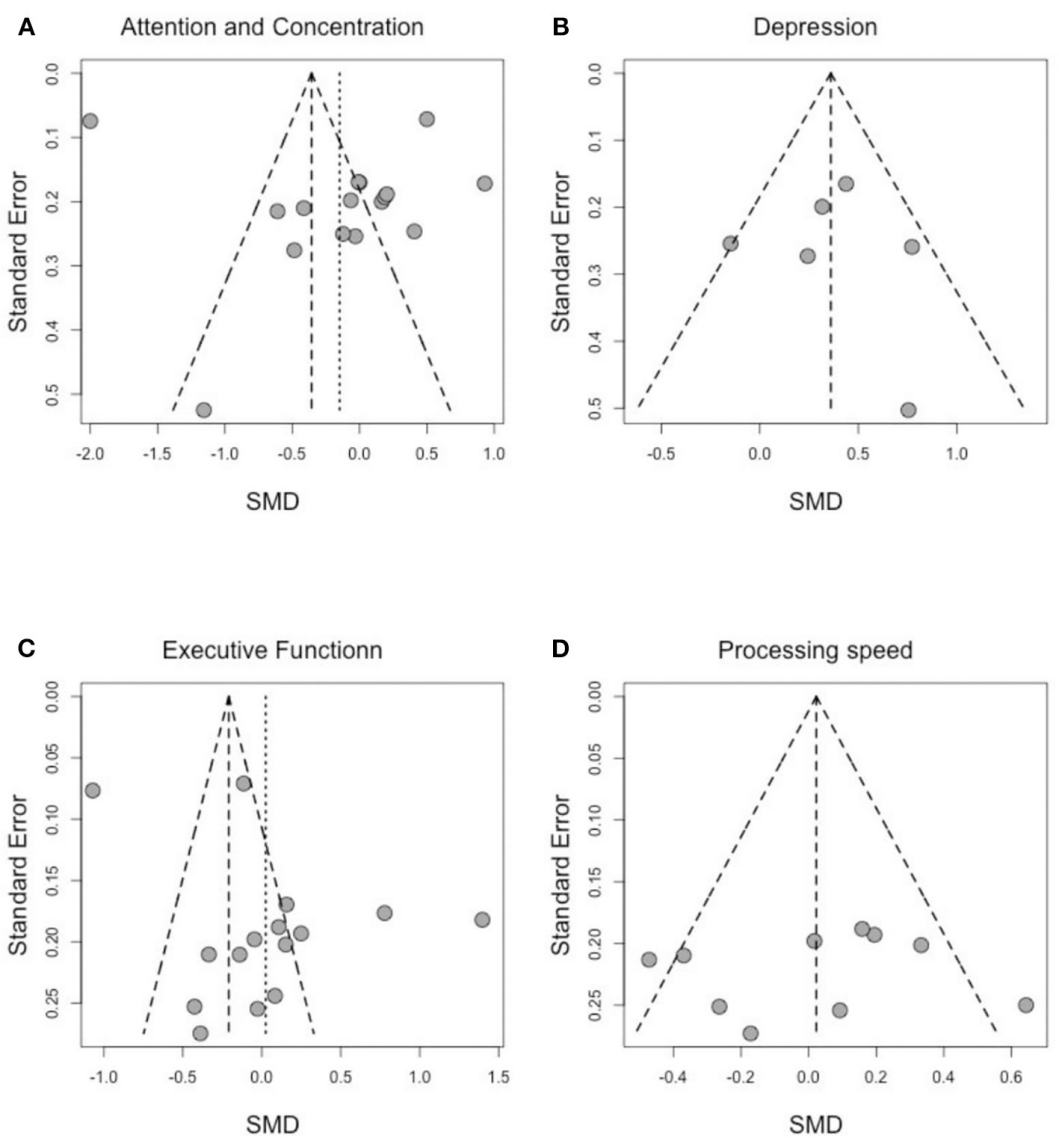
Funnel plot of effect sizes by SE for **(A)** attention and concentration, **(B)** depression, **(C)** executive function, and **(D)** processing speed.

To address publication bias, we included all studies regardless of the assessment time-points. Analyses were visually inspected using funnel plots, looking at asymmetry of the graph to assess publication bias across studies in the highly affected cognitive domains. As shown in Figure 4, assessment of the funnel plots of standardized mean difference (SMD) and the SE of effect sizes are symmetric around the overall weighted mean effect size in the domains of processing speed and depression, which indicates no significant publication bias.

We observe an asymmetry in the funnel plots for “attention and concentration” and “executive function.” However, Egger's tests are both not statically significant (Egger's test statistic: 0.99 *p*-value: 0.34 for attention and concentration; Egger's test statistic: 1.95 *p*-value: 0.07 for executive function) suggesting no asymmetry due to publication bias. As the publication bias was either not statistically significant (for attention and concentration, and for executive function) or not present (for depression and processing speed), no further adjustments were needed to account for publication bias.

## Discussion

The mechanisms of cognitive impairment in cancer survivors are not fully understood and are likely to be multifactorial. The current meta-analysis is the first to focus on the effect of taxane-based chemotherapy on various domains of cognitive functions. Any meta-analysis is limited by studies that are available, but we were able to identify 11 studies that evaluated post–taxane-based chemotherapy effects on cognitive functions in breast cancer survivors. Because these studies were selected using the term “taxane-based chemotherapy,” more than 800 (77%) of subjects with breast cancer included in our study received a taxane.

The present study adds to the literature in several ways. First, to our knowledge, it is the only study that focuses on the long-term (≥6-month post-chemotherapy completion) effects of taxane-based chemotherapy on cognitive functions in breast cancer survivors. This study was intentionally designed to minimize any effects of acute or stress-related changes in cognitive function. Cognitive functioning in the post-treatment period (i.e., 6 months or more post-therapy) was only discussed in one meta-analysis in 2012 (11). We limited our analysis to longitudinal studies, and excluded cross-sectional ones because we believe cross-sectional studies are not informative in elucidating etiology and the impact of chemotherapy on cognition (62). Second, our meta-analysis is based on data from studies that used objective testing of cognitive function, using validated and approved neuropsychological measures. We excluded studies that used subjective self-report outcomes or unvalidated methodology used for the assessment of cognitive function. Third, we excluded studies that included subjects more than 69 years old, as previous studies showed advanced age emerged as a significant moderator, indicating that older age at chemotherapy treatment was associated with poorer performance in neuropsychological measures (63).

The results of this analysis demonstrated that attention and concentration, depression, and executive function domains displayed significant chemotherapy-induced impairment at 6MPCC. A noticeable, but not significant, impairment was observed in the other domains of processing speed and visuospatial ability (Figures 3A,B; Tables 4, 5). Moreover, when neuropsychological testing scores of chemotherapy patients were compared with healthy control data, significant impairment was found in the effect sizes for four domains of cognitive function (executive function, information processing speed, and depression) (Table 4). This is consistent with previous meta-analyses that studied the effects of different therapy regimens on cognitive functions in cancer survivors (9–11, 64).

Although none of these prior studies focused exclusively on taxane chemotherapy, all of these previous meta-analyses reported significant impairments in multiple domains of functioning, most frequently executive function, processing speed, motor function, and memory. In a comparison of patients with different types of cancer, one of the largest longitudinal prospective clinical studies found that 43% of colorectal cancer patients showed cognitive impairment soon after diagnosis and before any chemotherapy, compared with 15% of non-cancer controls, and that 46% of survivors reported impairment 12 months later, compared with 13% of controls (65). In the present study, the rate of cognitive impairments at 6MPCC was significantly higher in those who received chemotherapy, although it has improved over time in some domains. The cognitive domains most affected included attention, verbal memory, and processing speed.

In 2019, a systematic review investigating cognitive impairment after cytotoxic chemotherapy examined 21 studies with enrolled patients with breast cancer (66). This report was not included in our analysis because it was not limited to taxanes but included other agents such as vinca alkaloids, anthracyclines, platinum compounds, topoisomerase inhibitors, and bleomycin and mitomycin (66). In addition, this report did not subdivide the cognitive changes into domains, but rather showed overall decreased cognitive function (66). Nonetheless, a similar pattern of CICI was reported compared with that which we observed.

It is curious that patients treated with chemotherapy often achieved *better* scores than the comparison group on some domains, suggesting that chemotherapy may give rise to some cognitive improvement. This observation is in accordance with the findings of one large-scale, longitudinal trial (67) that failed to identify any significant changes in cognitive function among chemotherapy patients, non-chemotherapy patients, and healthy controls. The investigators suggested that the inclusion of different treatment regimens was a possible factor for why cognitive impairment was not evident in the chemotherapy group. However, another study suggested that factors such as age and pretreatment cognitive reserve may be the reason for improved performance over time (45).

In the present analysis of breast cancer survivors treated with taxane-based chemotherapy, some aspects of cognitive functioning are slightly better after chemotherapy. Cognitive functioning showed improvement in the domains of verbal memory, visual memory, language, and visuospatial and motor function after 6 months or more of chemotherapy completion when compared with their own baseline assessments and when compared with breast cancer patients who did not receive chemotherapy (Table 4). These results may not imply that chemotherapy improves cognitive functioning or contradicts the adverse effects of chemotherapy on cognitive function. Instead, these improvements could be explained, at least in part, by the timing of post-treatment assessment. For example, breast cancer patients may have overcompensated for short-term cognitive impairment associated with chemotherapy and/or established compensatory intellectual strategies after undergoing multiple chemotherapy doses (43).

Some methodological limitations could also add to the explanation of this apparent improvement in post-chemotherapy cognitive functioning. For example, in all included longitudinal studies, patients' baseline cognitive functioning was measured just before initiation of chemotherapy. Beliefs around chemotherapy, patients' uncertainties about the effectiveness of treatment, and the extent to which they had been prepared for the experiences of chemotherapy could have a negative influence on patients who are recently diagnosed with breast cancer and guided to chemotherapy treatment (68). Another methodological explanation is the development of practice effects (PEs) on patients' performance at follow-up. This issue is a well-known characteristic in prospective longitudinal studies (69, 70). This suggestion was supported (46) by showing that when PE was not corrected, progressive improvement over time in measures of memory and divided attention was observed. Conversely, when PEs were corrected, worsening was found in measures of memory, fluency, executive function, and attention domains.

The microtubule network is critical for the formation and stabilization of spines, dendrites, and axons in all neurons, and this network is important for maintaining neurotransmission (71).

We hypothesize that taxanes can destabilize neuronal structure and impair neurotransmission. Specifically, alteration in hippocampal functions can provoke depression development. Taxanes can also lead to loss of spines and dendritic arborization. Consequently, loss of cortical gray matter will result in impaired cortex-based task performance, including attention, verbal memory, and executive functions (72).

There are few studies correlating the microtubule-stabilizing effect of paclitaxel with impaired memory acquisition in rodent models (72–74), although only some of these studies investigate effects on neuronal morphology. In addition, there have been other suggestions to use microtubule-stabilizing agents to normalize microtubule dynamics and to counter spine instability in neurodegenerative disease (75).

The results from the present analysis support the hypothesis that taxane chemotherapy is positively attributable as a risk factor for the development of chemotherapy-induced cognitive impairment. Findings from this study also showed that taxane chemotherapy was associated with a less favorable outcome regarding depression during and after treatment which can impact the lives of patients and their families. The findings were also discussed in previous literature that suggested that infusion therapies may be more distressing than oral therapies (76). This extensive impact justifies performing routine screening and monitoring of depressive symptoms in cancer survivors.

However, the present results do not support the hypothesis that taxane-based chemotherapy is the only cause of cognitive dysfunction in patients with breast cancer. The type of chemotherapy regimen, the use of different dosing and dosages, stage of disease, and cognitive reserve are all important contributing factors that may influence the cognitive outcome measures. Many of these factors could not be assessed in this meta-analysis due to lack of relevant studies. Investigators and healthcare professionals must become more aware that it is essential to identify individuals at high risk for cognitive impairment to enable intervention at an early stage. Training programs including in-person or online cognitive-behavioral therapy curricula can be used as a practical, accessible, inexpensive, and easily disseminated to breast cancer survivors as part of survivorship care plans.

### Limitations

As for any meta-analysis, this compilation has some limitations. It is possible that we have overlooked relevant studies despite our detailed search strategy. One possible source of overlooked studies is non–English language studies, which we specifically excluded. Due to lack of appropriate studies, we could not compare the effect on cognitive function of chemotherapy regimens that contained taxanes with those that did not contain taxanes. In addition, 77% of the included patients received taxane and it is challenging to state that taxane is the only cause of cognitive impairment. Therefore, individual patient data (IPD) meta-analyses will be considered in the future to address this issue.

There were methodological limitations in calculation of an average effect size of a certain domain; bias was introduced by the fact that data were pooled from different neurophysiological tests. This problem was also faced in the previous meta-analyses that discussed CICI and we used the same strategy they adopted in presence of multiple tests for same domain (10). Multivariate meta-regression would have allowed us to identify various demographic and disease-related factors that moderate the magnitude of post-chemotherapy cognitive impairment, but there were too few studies identified for this approach to be reliable. We did not include preventive interventions for CICI in our search terms, and therefore it was not possible to quantify the effectivity of preventive agents or approaches explicitly in our analysis.

## Conclusion

The effects of taxane-based chemotherapy on cognitive functioning among breast cancer patients were found to be variable, but relatively consistent with three domains being impaired, namely, attention and concentration, depression, and executive function. Certain cognitive domains (e.g., language and verbal memory) may show improvement over time as they may be more susceptible to practice effects.

There are several clinical and research implications of this meta-analysis. Clinically, our findings suggest that patients with breast cancer considering taxane-based chemotherapy need to be educated regarding the possibility of developing altered cognitive functioning 6 months after taxane treatment. Although the published data were limited, these changes appear to improve over time. In terms of research, the present study contributes to the scientific knowledge about the relation between breast cancer, taxane chemotherapy, and cognitive functioning. Further research is needed to clarify the role of different regimens and doses of taxane treatment, and the duration of the cognitive effects of chemotherapy among breast cancer survivors.

## Data Availability Statement

The datasets presented in this study can be found in online repositories. The names of the repository/repositories and accession number(s) can be found in the article/Supplementary Material.

## Author Contributions

EI and BE: Conception and design. KN, EI, ID, and ME: Collection and assembly of data. EI and ID: Data analysis and interpretation. All authors: Article writing and final approval of article.

## Conflict of Interest

BE is the founder of Osmol Therapeutics, a company that is targeting NCS1 for therapeutic purposes. SM receives consulting fees from Eisai Inc. and Celgene Corporation. She has received research funding from Pfizer (as joint funding between Pfizer and NCCN paid directly to her institution) and Genentech (paid directly to her institution). ID is a biostatistician at the West Haven, CT Cooperative Studies Program Coordinating Center, VA Office of Research and Development. The views expressed in this article are those of the author (ID) and do not necessarily reflect the position or policy of the Department of Veterans Affairs or the United States government. The remaining authors declare that the research was conducted in the absence of any commercial or financial relationships that could be construed as a potential conflict of interest.
