# Supplementary material for: Cognitive Effects and Depression Associated With Taxane-Based Chemotherapy in Breast Cancer Survivors: A Meta-Analysis
Source: Front Oncol. 2021 Apr 29;11:642382. doi: 10.3389/fonc.2021.642382 (PMC8121254; doi:10.3389/fonc.2021.642382)
Supplement: Supplementary file 1 [file Data_Sheet_1.docx]

| **Ovid MEDLINE(R) ALL <1946 to February 05, 2020>** | |  |
| --- | --- | --- |
| Search history sorted by search number ascending | | |
| **#** | **Searches** | **Results** |
| 1 | [Eiman Ibrahim search] | 0 |
| 2 | [cognitive function concept, without the broad searches for side effects] | 0 |
| 3 | exp Cognition Disorders/ | 90978 |
| 4 | exp Neurobehavioral Manifestations/ | 273991 |
| 5 | exp Mental Processes/ | 1019362 |
| 6 | Attention/ | 76114 |
| 7 | exp Neuropsychological Tests/ | 174214 |
| 8 | exp anxiety/ | 82369 |
| 9 | exp anxiety disorders/ | 78069 |
| 10 | exp cognition/ | 156020 |
| 11 | depression/ | 114878 |
| 12 | exp depressive disorder/ | 106839 |
| 13 | concentration.mp. | 1461441 |
| 14 | memory.mp. | 272511 |
| 15 | executive function.mp. | 23376 |
| 16 | (cognition or cognitive).mp. | 425664 |
| 17 | (forgetting or word finding or word retrieval or recall or object naming or action naming).mp. | 77985 |
| 18 | (CICI or CRCI).mp. | 142 |
| 19 | (depression or depressive or depressed).mp. | 493461 |
| 20 | anxiety.mp. | 227758 |
| 21 | hypervigilan*.mp. | 792 |
| 22 | (chemobrain or chemofog or chemo-brain or chemo-fog).mp. | 268 |
| 23 | (chemo* adj5 (fog or foggy or fogginess or brain)).mp. | 3736 |
| 24 | ((cognit* or neurocognit* or neuropsycholog* or neurobehavior* or neurobehaviour* or problem solving or attention or concentration) adj5 (deficit* or declin* or disorder* or function* or dysfunction* or disfunction* or impair* or decrement* or disturb* or problem* or sequelae* or change or changes or side effect* or loss* or improvement* or symptom or symptoms or effect or effects or complication*)).mp. | 468730 |
| 25 | cognitive.ti,ab,kf. | 345036 |
| 26 | (verbal fluency test or KWCST).mp. | 701 |
| 27 | (FACT-cog or headminder).mp. | 60 |
| 28 | or/3-27 | 3642061 |
| 29 | [population] | 0 |
| 30 | exp breast neoplasms/ or (breast adj6 (cancer* or neoplas* or carcinoma* or tumour* or tumor* or adenocarcinoma* or malignan*)).mp. | 392024 |
| 31 | [exposure] | 0 |
| 32 | exp paclitaxel/ or (anzatax or taxol* or paclitaxel or paxene or praxel or onxol or taxiter or taxotere or taxane*).mp. or P88XT4IS4D.rn. or exp taxoids/ or docetaxel/ or docetaxel.mp. | 55509 |
| 33 | [summation] | 0 |
| 34 | 28 and 30 and 32 | 921 |
| 35 | limit 34 to yr="1980 -Current" | 921 |
| 36 | limit 35 to english | 880 |
| 37 | exp children/ not exp adults/ | 1191253 |
| 38 | 36 not 37 | 880 |

| **Embase <1974 to 2020 February 18>** | |  |
| --- | --- | --- |
| Search history sorted by search number ascending | | |
| **#** | **Searches** | **Results** |
|  | | |
| 1 | [Embase Eiman Ibrahim search] | 0 |
| 2 | [cognitive function concept, without broad searches for side effects] | 0 |
| 3 | exp cognitive defect/ | 476062 |
| 4 | mental function/ | 13979 |
| 5 | exp cognition/ | 2188619 |
| 6 | neuropsychological test/ or exp cognitive function test/ | 92720 |
| 7 | anxiety/ | 197232 |
| 8 | exp anxiety disorder/ | 233340 |
| 9 | mental function assessment/ or exp cognition assessment/ | 25451 |
| 10 | exp depression/ | 459258 |
| 11 | memory.mp. | 380329 |
| 12 | executive function.mp. | 46732 |
| 13 | (cognition or cognitive).mp. | 652414 |
| 14 | (forgetting or word finding or word retrieval or recall or object naming or action naming).mp. | 94213 |
| 15 | (CICI or CRCI).mp. | 229 |
| 16 | (depression or depressive or depressed).mp. | 735244 |
| 17 | anxiety.mp. | 352008 |
| 18 | hypervigilan*.mp. | 1150 |
| 19 | (chemobrain or chemofog or chemo-brain or chemo-fog).mp. | 455 |
| 20 | (chemo* adj5 (fog or foggy or fogginess or brain)).mp. | 5590 |
| 21 | ((cognit* or neurocognit* or neuropsycholog* or neurobehavior* or neurobehaviour* or problem solving or attention or concentration) adj5 (deficit* or declin* or disorder* or function* or dysfunction* or disfunction* or impair* or decrement* or disturb* or problem* or sequelae* or change or changes or side effect* or loss* or improvement* or symptom or symptoms or effect or effects or complication*)).mp. | 625379 |
| 22 | cognitive.mp. | 556771 |
| 23 | (verbal fluency test or KWCST).mp. | 1373 |
| 24 | (FACT-cog or headminder).mp. | 162 |
| 25 | exp breast tumor/ or (breast adj6 (cancer* or neoplas* or carcinoma* or tumour* or tumor* or adenocarcinoma* or malignan*)).mp. | 609095 |
| 26 | [exposure concept] | 0 |
| 27 | paclitaxel trevatide/ or paclitaxel eluting coronary stent/ or paclitaxel/ or paclitaxel poliglumex/ or paclitaxel ceribate/ or paclitaxel derivative/ or paclitaxel tocosol/ | 104431 |
| 28 | taxoid/ | 2604 |
| 29 | docetaxel/ | 57081 |
| 30 | taxane derivative/ | 13247 |
| 31 | (anzatax or taxol* or paclitaxel or paxene or praxel or onxol or taxiter or taxotere or taxane*).mp. | 125871 |
| 32 | docetaxel.mp. | 58883 |
| 33 | or/27-32 | 156908 |
| 34 | [] | 0 |
| 35 | [] | 0 |
| 36 | [] | 0 |
| 37 | concentration.ti. | 83629 |
| 38 | concentration.kw. /freq=2 | 1144 |
| 39 | concentration.ab. /freq=2 | 462086 |
| 40 | or/37-39 | 509261 |
| 41 | (or/3-24,40) and 25 and 33 | 3022 |
| 42 | [summation] | 0 |
| 43 | limit 41 to yr="1980 -Current" | 3022 |
| 44 | limit 43 to english language | 2909 |
| 45 | exp child/ not exp adult/ | 1866670 |
| 46 | 44 not 45 | 2904 |
| 47 | limit 46 to (conference abstract or conference paper or "conference review" or conference proceeding) | 657 |
| 48 | 46 not 47 | 2247 |
| 49 | limit 48 to human | 2187 |
| 50 | limit 48 to animal | 32 |
| 51 | 48 not (50 not 49) | 2215 |
