# Supplementary material for: Cognitive Effects and Depression Associated With Taxane-Based Chemotherapy in Breast Cancer Survivors: A Meta-Analysis
Source: Front Oncol. 2021 Apr 29;11:642382. doi: 10.3389/fonc.2021.642382 (PMC8121254; doi:10.3389/fonc.2021.642382)
Supplement: Supplementary file 2 [file Data_Sheet_2.docx]

***Appendix two : Criteria for study population selection***

***Inclusion criteria:***

- Adult patients with breast cancer, at any stage, with the exception of patients with brain metastases
- Minimum age18 and maximum age 69.
- Patients who had received taxane treatment (alone or in combination with other treatments);
- Patients who were tested for any cognitive impairment following the taxane treatment of their breast cancer;
- Patients who completed standard-dose taxane at least 6 months prior to assessment of cognitive impairment. Six months after treatment was chosen as a cutoff point to exclude assessment of the acute effects of chemotherapy.

. ***Exclusion criteria:***

- Stage IV patients with brain metastasis will be excluded because of the direct effect of the tumor on the brain and consequently cognitive function;
- We will exclude research literature involving children, teenagers and adolescents.
